# Multiscale Cues Drive Collective Cell Migration

**DOI:** 10.1038/srep29749

**Published:** 2016-07-27

**Authors:** Ki-Hwan Nam, Peter Kim, David K. Wood, Sunghoon Kwon, Paolo P. Provenzano, Deok-Ho Kim

**Affiliations:** 1 Department of Bioengineering, Institute for Stem Cell and Regenerative Medicine, University of Washington, Seattle, WA 98195, USA; 2 Department of Electrical and Computer Engineering, Seoul National University, Seoul 151-742, Korea; 3 Division of Scientific Instrumentation, Optical Instrumentation Development Team, The Korea Basic Science Institute, Daejeon 34133, Korea; 4 Department of Biomedical Engineering, University of Minnesota, Minneapolis, MN 55455, USA; 5 Institutes of Entrepreneurial BioConvergence, Seoul National University, Seoul 151-744, South Korea; 6 Seoul National University Hospital Biomedical Research Institute, Seoul National University hospital, Seoul 110-744, South Korea; 7 Masonic Cancer Center, and Stem Cell Institute, University of Minnesota, Minneapolis, MN 55455, USA

## Abstract

To investigate complex biophysical relationships driving directed cell migration, we developed a biomimetic platform that allows perturbation of microscale geometric constraints with concomitant nanoscale contact guidance architectures. This permits us to elucidate the influence, and parse out the relative contribution, of multiscale features, and define how these physical inputs are jointly processed with oncogenic signaling. We demonstrate that collective cell migration is profoundly enhanced by the addition of contract guidance cues when not otherwise constrained. However, while nanoscale cues promoted migration in all cases, microscale directed migration cues are dominant as the geometric constraint narrows, a behavior that is well explained by stochastic diffusion anisotropy modeling. Further, oncogene activation (i.e. mutant *PIK3CA*) resulted in profoundly increased migration where extracellular multiscale directed migration cues and intrinsic signaling synergistically conspire to greatly outperform normal cells or any extracellular guidance cues in isolation.

Directional cell migration is essential for many normal and pathologic processes and is commonly regulated by gradients in chemical or physical factors and micro- and nano-structural patterns of the extracellular matrix (ECM)^1^,^2^,^3^,^4^. Indeed, the regulatory relationships between cell motility and ECM architecture are particularly important for proper glandular dynamics during development and adulthood^5^,^6^,^7^, and in the tumor microenvironment during invasion and metastatic dissemination^8^,^9^,^10^. For instance, during mammary gland development and in mature glands that undergo dynamic, hormone-regulated, expansion and regression, aligned collagen fibers radiate from the terminal end bud concomitant with invasion of epithelial cells into the mammary fat pad^5^,^6^. Likewise, during focal and local cancer invasion, ECM orientation plays a critical role in regulating cell migration^2^,^11^,^12^. Recent studies demonstrate that alignment of the stromal collagen matrix in murine mammary and human breast carcinomas drive invasion and predict poor outcome for human breast cancer patients^13^,^14^,^15^,^16^. In this context, groups of cells undergo collective invasion and interact with collagen fibers that provide microscale cues including constrained collective migration between fibers, and also single cells and sheets of cells interacting with individual fibers that present nanoscale cues from aligned collagen fibrils^6^,^13^. Furthermore, these findings are consistent with multiscale spatial cues from collagen fibers and fibrils that are present in developing and mature mammary gland dynamics^5^,^6^. Thus, in order to investigate how these complex multiscale guidance cues (i.e. microscale confined migration and nanoscale contact guidance) direct migration of normal and malignant epithelial cells, we engineered a biomimetic cell migration platform that allows precise control over these architectural parameters.

The effects of either ECM structure or specific oncogene activity on tumor cell motility are frequently investigated separately. However, the combinatory effect of these intra- and extra-cellular factors on the tumor cell motility and invasion has not been studied extensively and is not yet well understood. For instance, little is known regarding how specific oncogenic mutations, such as those of the phosphatidylinositol 3 kinase (PI3K) pathway that frequently occur in human breast cancer, influence how a cell interacts with the fundamental ECM architectures of the tumor microenvironment (TME). Interestingly, although studies have implicated *PIK3CA* mutations with features of transformation, e.g.^17^,^18^, definitive mechanisms describing how these mutations lead to changes in cell morphology, growth and motility have not been fully elucidated, but may identify new therapeutic targets associated with oncogenic *PIK3CA* mutations^19^,^20^,^21^. Here we seek to understand if aberrant PI3K signaling confers an advantage for cells migrating in micro/nano-environments conducive to invasion and metastasis (i.e. analogous to directed migration between collagen bundles and contact guidance along collagen fibers^6^,^13^,^22^). As such, we specifically introduce oncogenic activation of the PI3K pathway (mutation of *PIK3CA*, the p110α catalytic subunit of PI3K, frequently mutated in a number of different human cancers, including those of the breast^23^,^24^,^25^) in mammary epithelial cells interacting with complex physical cues.

Cell patterning on a defined substrate is a critical challenge so that fundamental questions about cell-cell and/or cell-substrate interactions and the systematic mechanisms driving their behavior in response to the local environment can be elucidated^26^,^27^,^28^,^29^,^30^,^31^,^32^. Precisely controlled cellular geometry can be achieved by engineering the surface properties of biocompatible materials to designate an arena for cell adhesion and motility. The main considerations to meet the demands of cell patterning techniques for the investigation of multiscale guided cell migration are that micropatterning technique should be not only compatible with nanostructural design and elastomeric substrate materials, but also its high-spatial resolution, the long-term stability of the patterns, and a homogeneous intensity as the patterns act as a migratory conduit with the defined various widths for precise control. To address these issues, plasma lithography^33^,^34^,^35^ has been utilized to investigate the cell migration in a defined microenvironment instead of many other promising techniques such as microscale plasma-initiated patterning^36^, microcontact printing^37^,^38^, the use of polyelectrolyte multilayers as an adhesion layer^39^, microelectrode arrays^40^,^41^, microfluidics-based methods^42^, inkjet printing^43^,^44^, and photolithography^33^. Plasma lithography creates surface modified geometry based on selective plasma surface functionalization of polymeric materials such as PDMS. Such modified geometry can be utilized to tune a wide range of mechanical and biochemical properties. The utility of this technique is most appreciated because of its long-term stability, reproducibility, scalability, and cost-effectiveness. Furthermore, nanotopography can be created on these materials using capillary force lithographic techniques^32^,^45^,^46^,^47^. Indeed, this method can be used to fabricate nanogroove patterns on elastomeric substrates, allowing for a wide range of applications due to cost effectiveness, scalability, and reproducibility^48^,^49^,^50^. Here, we utilize both of UV-assisted capillary force lithography and plasma lithography to fabricate various ECM micropatterns on nanotopography-featured elastomeric substrate for the purpose of providing non-transformed and transformed cells with contact guidance cues known to be operant in breast cancers.

To this end, we develop an engineered biomimetic cell culture platform that allows for the integrative study of complex stimuli, including micro and nanotopographic cues, and selective ECM architecture, to elucidate the roles of critical extracellular and intracellular factors and their interrelationship in the regulation of cell migration. We perform capillary force lithography and plasma lithography patterning on PDMS substrates with different geometries to guide and promote cell migration. Our results reveal that human breast epithelial cells and their genetic derivatives collectively sense and respond to the engineered microenvironment by integrating the microstructural and mechanical properties of the substrate during cell migration. Furthermore, we demonstrate that the biomimetic approach to modulate substrate-mediated cell migration may reveal essential factors that are responsible in phenotypic expression of oncogenic mutations. The results of this study signifies that topographic cues play an essential role in oncogenic activation and tumor cell metastatic behavior. Further investigations on the role of multiscale cues would be beneficial in understanding the underlying mechanisms of tumor metastasis and assist possible therapeutics development.

## Results

### Rational design and fabrication of an engineered biomimetic cell culture platform

**Figure 1:**
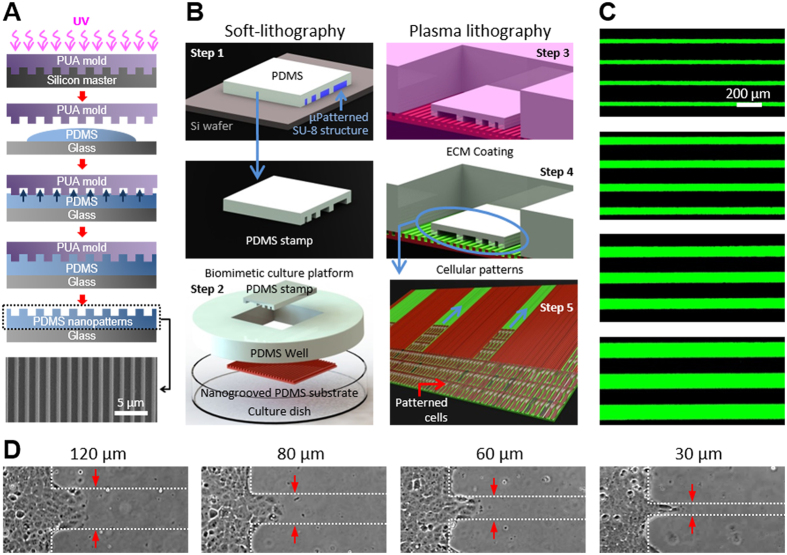
Fabrication of an engineered biomimetic culture platform. (**A**) Schematic diagram of the nanofabrication of topographically patterned elastomeric substrate using UV-assisted capillary force lithography. PDMS was drop dispensed onto coverglass, and then the coverglass was embossed with PUA master mold (800 nm ridge, 800 nm groove, 600 nm height). Capillary force lithography molded PDMS into the nanogroove pattern. Careful removal of the PUA master completed the nanofabrication. A SEM image of the nanogrooved substrate is shown at the bottom. (**B**) Schematic illustration of surface patterning using microstamp-assisted plasma lithographic techniques and ECM coating. A microchannel-patterned PDMS stamp was fabricated using soft-lithographic techniques and molding into PDMS (step 1). The cell culture platform was prepared for plasma lithographic patterning on the substrate (step 2). Oxygen plasma treatment (indicated by pink) of the device modified the originally hydrophobic nanogroove-patterned PDMS to hydrophilic, excluding the area in contact with PDMS stamp (step 3). Collagen type I at 50 μg/mL (indicated by green) was coated on the nanogroove-patterned PDMS for 6 hours (step 4). PDMS stamp was carefully removed after dispersed cells have reached monolayer. The removal of PDMS stamp resulted in a cell culture device with various widths of protein coated micropatterns (green surface) and collagen-untreated area (brown surface) (step 5). (**C**) Representative fluorescent images of the 30, 60, 80, and 120 μm width patterns of collagen type I (50 μg/mL) conjugated with Alexa Fluor 488-FITC (40 μg/mL). Only plasma-treated surface of the nanogrooved substrate was uniformly coated with the collagen type I solution even the inner parts of the grooves. (**D**) Microscopic images of patterned MCF-10A cells on nanogroove-patterned elastomeric substrates. After the removal of the PDMS stamp, the images were captured from the time point when the cells clearly entered 150 μm into the micropatterns for 12 hrs.

Three different lithographic techniques were applied to create spatial cellular patterns on nanogrooved elastomeric substrates. Namely, the engineered biomimetic cell culture platform was composed of three major parts; a nanogrooved elastomeric substrate fabricated by UV-assisted capillary force lithography and PDMS molding, a microchannel patterned PDMS stamp fabricated by soft-lithography, and selectively modified ECM patterns prepared by plasma lithographic process. The large area nanogroove pattern on a silicon master (2 inches by 2 inches) was successfully transferred to the thin PUA template fabricated by UV-assisted capillary force lithography. The SEM image shown in Fig. 1A (bottom) represents a complete nanofabricated PDMS substrate successfully cured over the PUA template. PDMS substrates were obtained from up to 50 replicas from a PUA template without any topographic defections. The thickness of the patterned PDMS substrates was less than 0.6 mm for microscopic observation. Consequently, the successful fabrication process of the large number of copies from a single template shows an inexpensive, mass producible, and reproducible nature of the capillary force lithography.

PDMS microstamp-assisted plasma lithographic techniques were used to create spatial surface patterns for MCF-10A culture on the nanogroove patterned elastomeric substrates (Fig. 1B, step 3). A PDMS microstamp was fabricated with photolithography to have nanopatterned substrate contact area (non-plasma treated) and open area (plasma treated). The open area (i.e. excluding the contact area with the stamp) was successfully modified and characterized by microscopic inspection of aqueous medium with dye. The restricted wetting confirmed a successful micro surface patterning. Here, two types of plasma lithographic patterns, straight-edged flat patterns as a control and 10 mm long line patterns with 30 (33 ± 1), 60 (60 ± 1), 80 (80 ± 2), and 120 (119 ± 2) μm width (15 lines for each dimension with 150 μm spacing) were created. The regular spacing between two micropatterns regardless of the width was suitable for preventing interrelation between migrating cells along each ECM-coated micropattern. This process was accomplished within 5 minutes and we confirmed that the technique provided long-term stability (>2 month) and high spatial resolution (>200 nm). Additionally, collagen type I was coated on the modified patterns (Fig. 1B, step 4) to enhance cellular attachment and movement by mimicking the primary ECM protein component of the tumor stroma. Successful surface modification and pattern structures were characterized by microscopic inspection of the collagen type I (50 μg/mL) with fluorescent dye wetted on only plasma treated areas as shown in Fig. 1C. Moreover, it was confirmed that the collagen was uniformly coated on the nanostructured surface including the inward regions of the nanogrooves by measurement of the cross-sectional fluorescent intensity of patterned microchannels.

**Figure 2:**
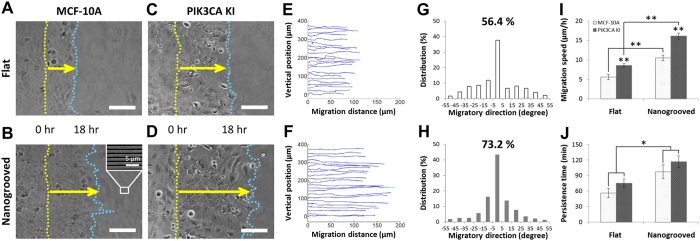
Migration of straight edge patterned cells on flat and nanogroove-patterned PDMS substrate. MCF10A wild type cells (**A,B**) and their mutant PIK3CA knockin cells (**C,D**) were patterned with a stencil after surface coating with type I collagen (50 μg/ml) on flat (**A,C**) and nanogrooved PDMS substrates (**B,D**). Scale bars = 100 μm, and an insert image (**B**) shows the direction of nanogrooves. Two images of straight-edged monolayers at 0 hr and 18 hrs respectively taken at the same positions combined into a single image. Yellow dot lines indicate the initial front edges of patterned cells and blue dot lines indicates the terminal front edges of migrated cells. Yellow arrows indicate a total migration distance over 18 hrs. The paths of individual migrating MCF-10A cells on flat (**E**) and nanogrooved (**F**) PDMS substrates were analyzed using custom MATLAB code. Individual migration paths of MCF-10A cells, defined as the angular deviation from the direction of nanotopography measured on both flat (**G**) and nanogrooved (**H**) substrates to assess migratory contact guidance. The portion of migration direction within ±15 degree from the nanogrooves representing straight directionality is highlighted in each graph. Migration speed (**I**) and persistence time (**J**) characterizing migratory responses of MCF-10A wild type and oncogenic PIK3CA knockin cells to substrate topography analyzed by fitting the mean-squared displacement of the cell path data to the persistent random walk model. Error bars represent standard deviations of three technical replicates with 80–100 cells per each experiment (*p < 0.01, **p < 0.001).

Breast epithelial cells were dispersed on the substrate enclosed by a PDMS well with an optimized seeding density of 1600 cells/mm^2^ for all experimental conditions (Fig. 1B, step 5). The dispersed cells were entirely trapped in a PDMS well allowing us to achieve the same seeding density on the patterned area. The PDMS stamp was removed when a complete monolayer was created to prevent the cells from settling on the ECM-coated micropatterns and lead the monolayered cells, not individual cells, to enter into the ECM-coated micropatterns at the same time as shown in Fig. 1D. The monolayered cells were selectively placed on the patterned area resulting in successful cellular pattern formation as the breast epithelial cells were confined to the plasma treated, and ECM coated, hydrophilic areas. The width of monolayered cells patterned at the left side shown in each microscopic image as well as the straight-edged flat patterns shown in Fig. 2A–D was designed 1 mm wide to diminish the effect of proliferation rate on directed cell migration. Further experiments were relatively short (much shorter than their doubling time of ~24–30 hrs^51^,^52^) so the effect of proliferation rate on migration speed was minimal and only non-dividing cells were tracked. Consistent with our validation using dye, the breast epithelial cells migrated along the line patterns and leave the patterns. Consequently, plasma lithography can efficiently modify nanogroove-patterned PDMS substrates. The nanotopography on the elastomeric substrate allows the cells to move in a directed fashion with the ECM patterns acting as geometric conduits while nanotopography provides contact guidance to direct migration direction and enhance migration speed of the cells. Therefore, the engineered biomimetic cell culture platform using the nanotopography and surface modification techniques allows independent control of geometric guidance and migratory trajectory to investigate directed migration of normal and transformed cells.

### Nanoscale contact guidance cues promote directed collective cell migration

Our previous studies demonstrate that substrate nanotopography allows cells to align along the nanogroove direction and promotes cellular attachment^45^,^53^. Here we quantitatively investigate the influence of multiscale cues in cells with and without an intrinsic perturbation (i.e. oncogenic mutation). First, straight-edged monolayers (1 mm wide, 14 mm long) of the breast epithelial cell line MCF-10A and its derivative, knockin of mutant *PIK3CA*, were generated on the ECM-coated elastomeric substrates using stencil-assisted plasma lithography as shown in Fig. 2. When the cell monolayer was created, the stencil was removed and image analysis confirmed the presence of wide straight-edged monolayers of either MCF-10A or *PIK3CA* knockin cells on flat substrates (Fig. 2A,C) or on nanogrooved PDMS substrates (Fig. 2B,D). Second, subsequent analysis of cell migration of both cell types demonstrates that the cells follow a persistent random walk model^54^,^55^,^56^ parameterized by cell migration speed and directional persistence time^57^,^58^, defined as the average time between significant changes in the direction of a cell’s translocation^54^. The speed of each cell was determined by dividing the root mean-square displacement (MSD) of the path of each migrating cell, tracked for *N* sequential positions by the constant time interval ∆*t*, calculated using the method of non-overlapping intervals^59^. Each cell’s persistence time was fit using nonlinear least-squares regression by inserting its speed into the persistent random walk model. The paths of individual cells migrating on both flat and nanogrooved substrates were analyzed over 18 hours with interval time-lapse imaging (*N* = 54, ∆*t* = 20 min) by tracking individual cell positions within each image.

Quantitative analysis of the clear qualitative differences in cell migration (Fig. 2A–D) demonstrates a significant difference in migration trajectories (migration distance and direction) of MCF-10A cells on flat (Fig. 2E) versus nanogrooved (Fig. 2F) PDMS substrates. Similar findings were observed with mutant *PIK3CA* cells (Fig. 2C,D). Furthermore, results suggest that human breast epithelial cells on nanogrooved substrates exhibited more linear migration trajectory with longer migration distance within a defined experimental time (Fig. 2F) than on flat substrates (Fig. 2E) indicating a strong contact guidance mediated migration along the direction of the nanogrooves. Likewise, migratory direction of individual paths defined as the angular deviation from the fabricated nanogroove direction was measured (Fig. 2G,H). The angle represents the degree by which cells deviate from the long axis of the nanogroove with 0 degree indicating that the direction of migration is in complete congruity to the direction of nanogrooves. The proportion of migration paths that were within ±15 degrees from the nanogrooves was calculated to specifically assess an effect of the nanogrooves on migratory contact guidance. From this analysis, we find that 56.4% of migration paths on the flat substrate and 73.2% on the nanogrooved substrate are within ±15 degree of the primary axis. Thus, nanotopographic features within microscale constrained migration regions further promote directed cell migration, with the addition of nanogrooves contributing significantly to contact guidance by producing an ~30% increase in directional migration compared to migration on flat substrates.

In order to further elucidate the influence of nanotopography on directed cell migration, we fit the mean-squared displacement of the cell path data to the persistent random walk model, as described above, to obtain migration speed (Fig. 2I) and persistence time (Fig. 2J). Interestingly, both the MCF-10A wild type cells and mutant *PIK3CA* knockin cells migrate on average 87 (±2)% faster (p < 0.001) along nanogrooved substrates than on flat substrates suggesting that encountering topographic nanogrooves results in altered motility dynamics to enhance migration speed. Moreover, oncogenic *PIK3CA* knockin cells migrate on average 53 (±2)% faster (p < 0.001) on both flat and nanogrooved substrates than their wild type counterparts, demonstrating that oncogenic mutations of PI3K enhance cell migration, consistent with the concept that *PIK3CA* may promote breast cancer metastasis. Additionally, directional persistence of both cell types (Fig. 2J) in the presence of nanoscale guidance cues showed a significant increase (p < 0.01), providing further evidence that contact guidance architecture enhances directed migration through increased persistence. Hence, these result show that the cells on nanopatterned substrates migrate for longer average time without significant changes in the direction, and with enhanced speed, than on flat substrates due to a straightforward contact guidance mediated migration along the direction of the nanogrooves.

### The width of microscale geometric constraints influences directed cell migration

**Figure 3:**
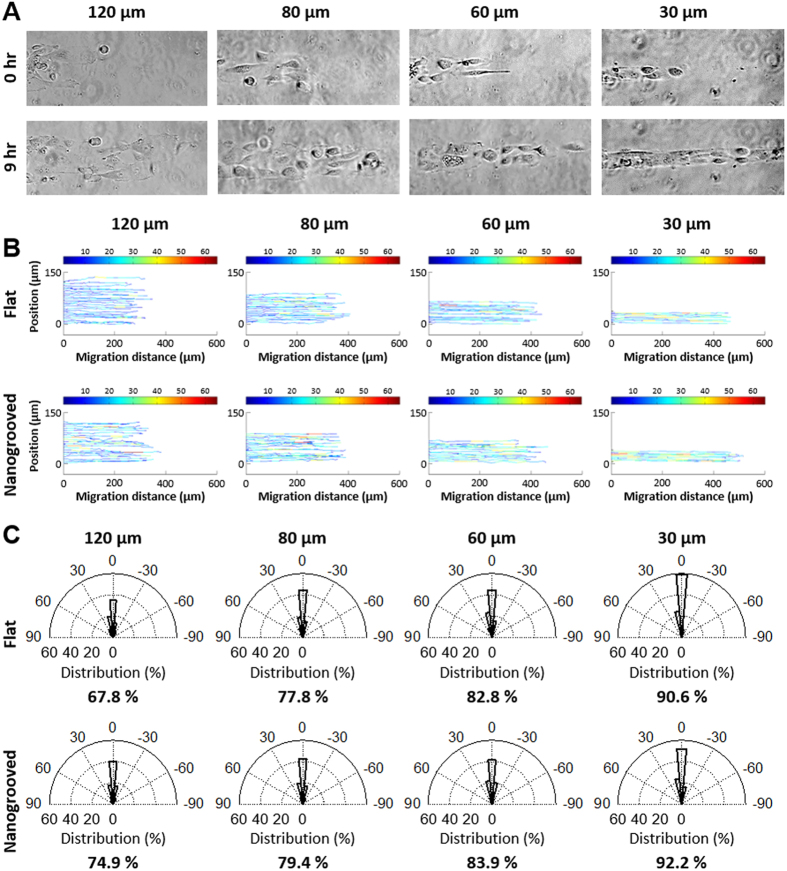
Cell migration on ECM patterned substrates using plasma lithographic techniques. (**A**) Microscopic images of mutant PIK3CA knockin cells migration on collagen type I patterned nanogrooved PDMS substrates. Time-lapse video microscopy was used to capture the cellular motility and digital images were taken every 20 min for a total of 12 hrs per experiment. Scale bars = 100 μm. (**B**) The paths of individual migrating PIK3CA knockin cells on flat (B top) and nanogrooved (B bottom) migration on PDMS substrates was analyzed using custom MATLAB code. Color bar legend indicates migration speed of cells at each time-lapse in units of 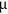
m/hr. (**C**) The migration direction of individual paths of PIK3CA knockin cells on both flat (C top) and nanogrooved (C bottom) substrates were measured and the portion of the migration directions within ±15 degree from the ECM patterns representing straight directionality of the migration is shown in each graph. As the ECM pattern widths became narrower from 120 μm to 30 μm, motile cells show greater straight directionality on both substrates with a decreased effect of nanotopography on migratory contact guidance.

Our experimental culture platform presented here not only promoted increased cell migration speed and persistence though nanoscale contact guidance cues, but also facilitated identification of significant differences in migration as a function of varying microscale geometric constraints. To further explore the influence of multiscale cues imparted by the ECM, elastomeric substrates were created with different microscale pattern widths by using PDMS microstamp-assisted plasma lithography to create spatial matrix patterning in order to mimic characteristic heterogeneity found within the normal and diseased *in vivo* tissue environment^60^. Along these lines, 10 mm long straight line collagen coated patterns with 30, 60, 80, and 120 μm widths were designed to elucidate the influence of the microscale spatial cue in the width direction. Cells were selectively patterned as described above and migrated only on the micropatterned ECM structures with defined geometry. Consistent with our previous findings (Fig. 2), the cells migrated only within the ECM-coated micropatterns without a remarkable difference in migration speed between cells in the middle and at the edge of the patterns, and did not go off the patterns during experiment. For quantitative analysis of cell migration, the paths of individual migrating *PIK3CA* knockin cells on flat and nanopatterned PDMS substrates along the spatial ECM patterns were tracked for 12 hours with time-lapse imaging (*N* = 36, ∆*t* = 20 min) to measure trajectories including migration direction and distance (Fig. 3B). The results demonstrate that as the pattern width become narrower (from 120 μm to 30 μm), the total migration distance of the cells along the patterns for all conditions becomes longer.

**Figure 4:**
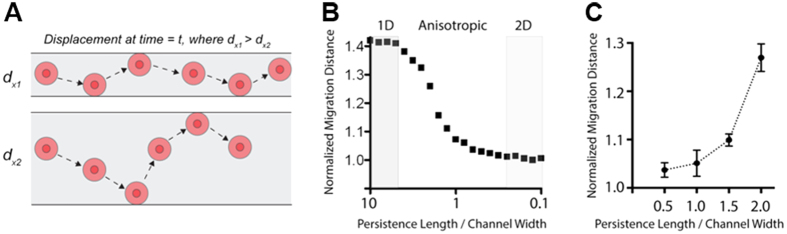
Directed migration from microscale geometric constraints conforms to diffusion anisotropy behavior. (**A**) Example of single particle (representing cells) undergoing a random walk in a geometrically constrained environment that results in increased motility coefficients, and thus mean displacements, in the x direction for a population of cells. Note, cells behind a collective migration front and lateral cell interactions further constrain the system. (**B**) Model output of normalized migration distance in the x direction over of a range of displacement steps with defined persistence length for a range of channel/pattern widths. Model output is from 100,000 steps/particle averaged over 1000 particles and data is normalized to random walk behavior in the unconstrained 2D configuration. (**C**) Normalized migration distance for four pattern widths highlighting the increase in migration along the x-direction as the geometric constraint narrows. Data are from the average of 10 runs with 300 particles undergoing 100,000 steps/particle.

This observed increase in migration distance as pattern width becomes narrower is consistent with confined anisotropic diffusion behavior and as such the observed changes in cell migration behavior as a function of pattern width is well explained by a random walk diffusion anisotropy model. We developed a stochastic model where cells undergoing a random walk are constrained by boundaries (i.e. pattern or channel width and cells behind the leading edge). As expected, as the channel width becomes much larger than the migration persistence length the model predicts classic random 2D migration behavior for cell undergoing a random walk (Fig. 4A bottom). Likewise, as the width becomes relatively small the output approaches a purely 1D directed migration regime (Fig. 4A top). Here, the model predicted that as channel width decreased the migration distance would vastly increase, which is consistent with experimental data (see Fig. 4C for an example of shifting the geometric constraint by a factor of 4). As such, model predictions strongly suggest that our observed increase in cell migration by confined microscale directed migration cues is the result, at least in part, of a thermodynamically driven random motility process directed by the geometrically constrained environment.

### Superimposed multiscale extrinsic guidance cues and cell intrinsic cues conspire to promote migration

To define the collective impact of micron scale constrained migration cues, nanoscale contact guidance cues, and intrinsic cellular perturbations, we generated patterns of varying widths possessing flat or nanogrooved surfaces and quantified the response to the cues in parental and *PIK3CA* MCF-10A lines. Selectively patterned cells migrated only on the micropatterned ECM with no remarkable difference in migration speed between cells in the middle and at the edge of the patterns consistent with earlier experiments. The nanotopography dimensions designed to provide contact guidance on the biomimetic cell culture platform were 800 nm ridges, 800 nm grooves with a height of 600 nm (see Figs 1 and 2), and are within the range fibril spacings observed in mammary collagen^6^. For quantitative analysis of cell migration, the paths of individual migrating *PIK3CA* knockin cells on flat and nanogrooved PDMS substrates along the spatial ECM patterns were tracked with time-lapse imaging (*N* = 36, ∆*t* = 20 min over 12 hours) to measure trajectories including migration direction and distance (Fig. 3B,C). Consistent with our results for flat substrates (Fig. 2E,F), analysis demonstrated that as the pattern width become narrower (from 120 μm to 30 μm) for a nanogrooved PDMS substrate, the total migration distance of the cells along the patterns for all conditions becomes longer (Fig. 3B). Note the migration speed also increases as the pattern width become narrower (Fig. 3B), as indicated by the heat map (blue, 0 μm/hr to red, 65 μm/hr). Furthermore, we note that the addition of nanoscale contact guidance cues within microscale-constrained patterns promotes collective migration, albeit quite modestly, particularly relative to the robust increase in migration observed in straight-edged monolayer results (i.e. Fig. 2). Thus, to further parse out the influence of nanoscale contact guidance cues within each constrained ECM pattern (30–120 μm widths) the direction of individual migration paths were quantified and the percentage of knockin cells with migration directions within ±15 degrees from the ECM patterns, representing highly persistent directionality of the migration, were analyzed (Fig. 3C). The result demonstrates that as the width of ECM patterns become narrower from 120 μm to 30 μm, on both substrates, migrating cells display an increase in directionality along the length of the pattern, consistent with prediction from the diffusion anisotropy model (Fig. 4A). However, although nanotopography enhances directed migration for each condition, the increase in the straight directionality between flat and nanogrooved substrates when the width of ECM patterns become narrower than 80 μm is quite modest (Fig. 4B).

**Figure 5:**
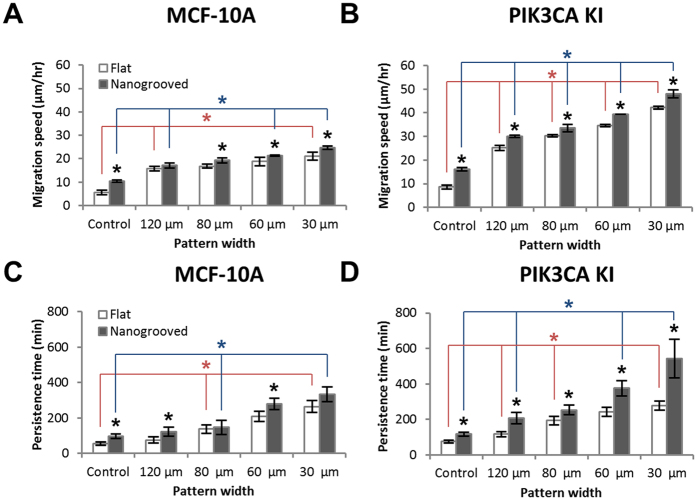
Persistence random walk model parameterized by cellular migration speed and directional persistence time. Cell migration speed of MCF-10A wild type cells (**A**) and oncogenic PIK3CA knockin cells (**B**) responsive to substrate nanotopography, and on flat and nanogrooved substrate to cell type based on ECM pattern geometry. Persistence time of MCF-10A cells (**C**) and oncogenic PIK3CA knockin cells (**D**) on ECM patterned flat and nanogrooved PDMS substrates were calculated by fitting the mean-squared displacement of cell path data to the persistent random walk model. Error bars represent standard deviations of three technical replicates with 40–50 cells per each experiment and statistical significance is indicated by P values (*p < 0.05).

To further explore the influence of ECM cues and contemporaneous intrinsic cues we compared parental and *PIK3CA* cells within patterns of different width contained flat or nanogrooved patterns. By calculating MSD of the cell path of each migrating cell, we obtained migration speed and persistence time from the persistent random walk model for both cell populations subjected to each ECM condition (Fig. 5). Consistent with the previous results (Fig. 2), the migration speed and the persistence time of both cell types varied with the pattern width (Fig. 5A,B), where decreasing pattern width resulted in increased migration speed. Yet, while nanoscale contact guidance cues again promoted migration speed, the impact was modest for both cell types in narrow patterns (Fig. 5A,B), suggesting that microscale directed migration cues are more dominant for both cell types as the pattern width approaches a few cell diameters. Indeed, in contrast to wide channels (control groups), both wild type and knockin cells on nanogrooved substrates migrated a modest 0.14 (±0.03) times faster on average than on flat substrates for all pattern widths (≤120 μm) but do display a significant (p < 0.05) or trending (0.05 < p < 0.07) increase at each pattern width supporting the conclusion of a synergistic influence of micro and nanoscale cues. The difference in migration speed between on flat and nanogrooved substrates at each pattern width, regardless of the cell type, was from at most 3.1 (±2.1) μm/hr to 3.8 (±1.6) μm/hr as the pattern width was decreased from 120 μm to 30 μm. In contrast, the impact of microscale directed migration cues resulted in a more substantial increase in migration where the migration of MCF-10A and *PIK3CA* knockin cells under control condition (i.e. relatively wide patterns) shifted from 5.6 μm/hr and 8.6 μm/hr respectively, to >20 μm/hr and >40 μm/hr at 30 μm ECM pattern widths. Yet, cell migration along the ECM patterns on both substrates was up to 5.6 times faster due to the synergistic effect of substrate nanotopography and surface patterning compared to conditions that lack the nanotopography and ECM micropatterns. Furthermore, migration speed of the oncogenic *PIK3CA* knockin cells on both flat and nanogrooved substrates were 97% faster than their wild type cells on average (Fig. 5A,B). Consequently, our data demonstrate that the oncogenic breast epithelial cells more sensitively respond to ECM patterns in a geometry-dependent manner, and there is a statistically significant difference (p < 0.05) in migration speed among the two cell types on all ECM patterns (Fig. 5A,B).

The persistence time also varied with the presence of nanotopography and ECM micropatterns of various widths. Increases in directional persistence of MCF-10A wild type cells in the presence of nanogrooves were modest but showed a statistically significant or trending difference at each pattern width, consistent with migration speed data (Fig. 5C). In stark contradistinction, the persistence time of oncogenic *PIK3CA* knockin cells in the presence of the nanogroove contact guidance cues showed a strong significant increases as well as a dependence on the pattern widths, rising from ~207 min at 120 μm to ~543 min at 30 μm showing profoundly straightforward movement without any significant turns during experiment (Fig. 5D). These findings demonstrate that oncogenic *PIK3CA* knockin cells migrate for longer time without significant changes in their migratory direction within the ECM micropatterns compared to their wild type cells resulting in an increased straightforward directionality. These results likewise provide further evidence that geometric confinement of migratory conduits modulates directional migration, and show that oncogenic *PIK3CA* knockin cells are more sensitive to the pattern geometry and the substrate nanotopography. Thus, in the oncogenic cell populations a profound synergy is observed between extracellular micro and nanoscale directed migration cues and intrinsic cell signaling that drives migration in a way that vastly outperforms wild type cells or any of the extracellular guidance cues in isolation.

## Discussion

In summary, we engineered a biomimetic cell culture platform using plasma lithography patterning and nanopatterning with UV-assisted capillary force lithography in order to provide multiscale directed migration cues and guide cell migration. This system facilitates the exploration simultaneous of extracellular and intracellular perturbations and the parsing out of their relative impact. Data here demonstrates that the plasma lithographic technique is an effective approach for creating geometric surface patterns based on selective plasma functionalization of the substrate with long term stability that effectively allow for the addition of nanopatterning. Thus this platform provides a highly effective and reliable means to expose epithelial cells to micron and nanoscale directed migration cues. Furthermore, although we have focused our studies here on mammary gland biology and the breast cancer micro/nano-environment, the versatile nature of these designs can be utilized to explore the behavior of many different cell types or mimic other physiologically relevant environments such as blood vessels, myelinated fibers, or the white matter tracks that glioma cells in the brain have been shown to migrate along. Likewise, the integrative nature of our system, and the ability incorporate different surface chemistries, could be employed to investigate additional factors such as the synergistic effects of protein ligand mediated migration with topographic guidance. It is also envisioned that other mechanical factors, such as mechanical deformation, gradients in substrate stiffness, fluid shear stress, and chemical gradients will be incorporated into the platform to further decipher complex behavior. Thus, we present a robust platform that can be employed in its current form, or easily adapted, to address fundamental questions in how the environment influences cell motility as a function of the underlying genetics and associated proteome.

In our experiments, with straight-edge pattern cells (i.e. effectively very wide patterns; Fig. 2), contact guidance from nanoscale cues was prominent, giving rise to significantly increased speed and persistence time with straightforward directionality in the direction of the nanogrooves versus migration on a flat surface. In addition, the nanoscale contact guidance cues resulted in increased directionality of migration, collectively resulting in vastly enhanced distance of migration within a set time window. Furthermore, through this platform we were able to identify microscale directed migration cues that also profoundly influence collective cell migration. As pattern width (analogous to channel width) decreased to 120 μm cell migration increased robustly (i.e. Figs 3 and 5) compared to wide environments (Fig. 2 and control groups in Fig. 5). Furthermore, as width was decreased from 120 μm down to 80, 60, and ultimately 30 μm cell migration significantly increased, a behavior consistent with diffusion in a constrained environment (i.e. diffusion anisotropy for cells undergoing a random walk). Interestingly, these results contrast findings in Madin-Darby canine kidney (MDCK) cells in channels spanning 100 to 300 μm 61, but are consistent with increased migration of MDCK cells as pattern widths decrease from 400 down to 100 and then particularly at 20 μm 62. Combined these results and our findings reported here then suggest that directed migration from microscale constraints is most operant when the constraint is in a range below approximately 100 μm in width. Of course, however, this dimension is relative to cell size and the persistence length of the migrating cell, as supported by our diffusion anisotropy model. We also note that the addition of nanoscale contact guidance cues, while substantially increasing migration in wide patterns, display a much more modest improvement as the pattern width decreases. Thus, it is of interest to note that these cues (microscale and/or nanoscale) may be thought of as drivers to dictate profoundly different diffusion coefficients (i.e. motility coefficients in this context) in different directions as is readily illustrated by considering Fick’s law diffusion behavior in multiple dimensions where the diffusive flux (*J*_*Diff*_) is represented as


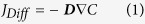


with cell concentration *C.* Considering multiple directions, the diffusion coefficient *D* may be non-isotropic as represented by a symmetric tensor to account directional dependence of the diffusion coefficient, i.e.


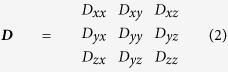


where for the 2D problem this would reduce and account for anisotropy in the x and y directions. This is analogous to previous work describing directional dependent random walk^63^ and biased migration through an anisotropic diffusion parameter proportional to the ratio of the diffusion coefficient in two orthogonal directions (e.g. x and y) that can account for directed migration effects, including contact guidance^64^. This approach was recently corroborated to describe separate cell types undergoing 3D migration in nonhomogeneous environments^65^. Likewise, we note that the additive effect of nanoscale contact guidance cues to further promote migration within confined micropatterns (i.e. *Fickian diffusion*) that enhance migration may be conceptualized in this context as a convective driving force (***J***_***conv***_ = *C**v***, where ***v*** is velocity) additive to the diffusive flux behavior


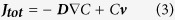


In this context the convective driving force would account for fundamental changes in the propulsion force resulting from alterations in the mechanics of cell migration resulting from contact guidance architectures. Furthermore, the ratio of these terms may have implications for characterizing the dominant cues through a dimensionless parameter, as would be the case for dominant micron scale directed migration cues in narrow channels (e.g. Figs 3 and 5), but much less so for the case of effectively wide channels (e.g. Fig. 2) where nanoscale contact guidance cues impart a much more substantial increase in migration.

PI3K pathway signaling regulates numerous critical behaviors required of transformed cells, including growth, survival and invasion^19^,^20^, and it is frequently dysregulated in numerous malignancies^23^,^24^,^25^, making it an attractive target for cancer therapy. Further, in addition to therapeutic exploitation of its well-described downstream effectors (e.g. AKT, mTOR etc.), characterizing its motility dynamics in response to particular features found in the TME may open new avenues to identify novel therapeutic vulnerabilities in PI3K mutant cells. Here, we demonstrate that mutant *PIK3CA* expression resulted in significantly increased cell migration versus parental MCF-10A cells under baseline (i.e. wide and flat) conditions. In addition, exposure to nanoscale contact guidance cues significantly promoted migration of mutant cells. What is perhaps surprising is that parental MCF-10A cells showed only modestly increased migration with the addition of nanoscale contact guidance cues in the context of narrow patterns microscale geometric constrains. In contrast, oncogenic cells display a remarkable synergy where the additive benefits of microscale, nanoscale, and PI3K signaling are realized. As a result, these cells vastly outperformed wild type cells under any of the directed migration cues, suggesting that oncogenic breast epithelial cells are more heavily influenced by multiscale directed migration cues in the environment. These observations suggest that as the pattern width diminishes, microscale directed migration cues become more dominant, appearing to exceed the influence of nanotopography induced contact guidance for collective cell migration in this context. Indeed these results suggest that the activation of a single oncogene may confer a distinct advantage for migration through a given ECM architecture and this response may correlate the metastatic potential of carcinoma cells. Further, these finding support the conclusion that oncogene activation in this context resulted in a fundamental change in adhesion-mediated signaling and the dynamic cytoskeletal remodeling underlying sustained locomotion through persistent protrusion of leading edge lamellipodia over time. Yet, the exact processes by which these oncogenic cells display vastly increased persistence, as well increased speed, to profoundly increase migration remain to be elucidated. Certainly, clarifying the interrelationships between the multiscale directed migration cues presented here and the underlying intrinsic cell structure and signal transduction in a quantitative manner will add considerably to our understanding of the fundamental mechanisms regulating normal and cancerous cell migration.

## Materials and Methods

### Fabrication of nanotopographically-defined cell culture substrates

To prepare topographically nanogroove-patterned (nanogrooved; 800 nm width, 600 nm height with 800 nm spacing) elastomeric substrate, a mold made of polyurethane acrylate (PUA) was fabricated from a silicon master patterned via e-beam lithography (JBX-9300FS, JEOL) including photoresist development (MF320, Shipley), deep reactive ion etching (STS ICP Etcher), and ashing process (BMR ICP PR Asher) as previously described^51^,^52^. Successively, as shown in Fig. 1A, UV-curable PUA was dispensed onto the silicon master and the polyethylene terephthalate (PET) film was brought into contact with the dropped PUA solution followed by UV curing (MT-UV-A21, Minũta Technology) for 15 seconds. The PUA mold (thickness: 0.5 mm) was peeled off from the silicon master and additionally cured overnight. The topographically nanogrooved PDMS substrate was then fabricated using capillary-molding techniques^28^,^45^. Mixed PDMS solution was dispensed on the PET film and the PUA mold was directly placed onto the surface. The PDMS solution filled the cavity of the mold by means of capillary force and was cured at 75 °C for 100 min. After curing the mold was peeled off from the PDMS substrate.

### Microstamp-assisted plasma lithography for spatial cell patterning

The process of making cellular patterns on the substrate is composed of two different lithographic techniques. Photolithographic and plasma lithographic techniques were used to fabricate a microchannel-patterned PDMS stamp and create selective hydrophilic micropatterns, respectively. Thus creating a hydrophilic and hydrophobic array allows for cell adhesion on alternating lines (Fig. 1B). First, a microchannel-patterned stamp was fabricated using soft-lithographic techniques and molding into PDMS as described previously^66^,^67^. Briefly, the micropatterned template was prepared by fabricating a SU-8 master composed of various microchannels using standard soft-lithography techniques. A Si wafer was coated (Solitec spin coater) with photoresist (SU-8, MicroChem, MA), exposed to UV light (ABM mask aligner) through a chrome mask (Fineline Imaging, CO) to polymerize the photoresist, and finally the unexposed photoresist was washed away. 10 to 1 mixed PDMS prepolymer was dispensed onto the silicon master and then cured for 2 hours at 75 °C thereafter. Then, the 3D patterned PDMS stamp (14 mm (L) × 10 mm (W) × 2 mm (H)) was peeled off from the silicone master (step 1 in Fig. 1B).

Next, the surface of the substrates were modified using plasma lithographic techniques^35^,^60^ with the 3D microchannel patterned (20, 40, 60, and 100 μm width straight lines, 15 lines each) stamp to make spatial cellular patterns and to direct the cellular migration as shown in Fig. 1B. First, a culture platform was composed of the 3D micropatterned PDMS stamp, a PDMS barrier used for trapping cells, a topographically nanogroove-patterned PDMS substrate, and a coverglass. The PDMS stamp was placed in conformal contact with the surface of flat and nanogrooved substrates surrounded by a large PDMS well (step 2 in Fig. 1B). The cell culture platform was then exposed to atmospheric plasma (CUTE, Femto Science) set at 80 W and 0.5 Torr for 3 min (step 3 in Fig. 1B). Due to the physical channels formed between the 3D stamp and the substrate, the substrates were selectively and chemically modified by the plasma, creating micropatterns that serve as the only passageways for cell migration. To improve attachment, movement, and mimic the primary protein ECM component of the tumor stroma, collagen type I (50 μg/mL) solution was subsequently injected into the large PDMS well (14 × 12 × 8 mm), which was made to trap cells only on the patterned area. The microchannels were fully filled with the injected ECM solution within 5 min, and the culture platform was then incubated. After 3 hours of incubation, the filled ECM solution was removed by air blast, and the microchannels were washed with 1 × PBS twice followed by air drying (step 4 in Fig. 1B). Finally, 0.2 mL of culture media was added into the PDMS well, and after the microchannels were filled with the media, cells were seeded into the well with defined seeding density. After one day of incubation, the 3D stamp was removed, and the culture platform was incubated for an additional 6 hours until the monolayered cells at both ends of the patterns created 150 μm extended arms of cells into the patterns (step 5 in Fig. 1B). Cellular migration along the micropatterns was imaged using a live cell imaging microscope for 18 hours.

### Cell culture and targeted knockin of the PIK3CA oncogene

All studies in this article were performed using stable lines of human-derived MCF-10A breast epithelial cells and MCF-10A mutant *PIK3CA* knockin cells (kindly provided by Prof. Ben Ho Park, The Johns Hopkins University). Culture procedures for the MCF-10A line and its derivatives has been described previously^19^,^20^,^21^. Briefly, MCF-10A cells and the *PIK3CA* knockin cell line were propagated in Dulbecco’s Modified Eagle’s Medium (DMEM)/F12 (1:1) (Life Science) supplemented with 5% horse serum, 20 ng/mL EGF, 10 g/mL insulin, 0.5 g/mL hydrocortisone, and 0.1 g/mL cholera toxin. All supplements were purchased from Sigma-Aldrich, unless otherwise noted. To generate the *PIK3CA* knockin line, targeting vectors were designed to introduce a single oncogenic mutation within *PIK3CA*. Vector transduction, colony selection, clone screening, and Cre recombinase removal of the neomycin resistance gene have been described previously^68^,^69^. For cell patterning, cell suspensions were introduced into the device at volumetric cell densities sufficient to achieve a subconfluent density, tested from 1300 cells/mm^2^ to 1800 cells/mm^2^, and then cells were allowed to adhere to the flat or nanogrooved elastomeric substrates. Prior to the start of imaging and data acquisition, cells were additionally cultured with a flat stencil or a microstamp to create a dense cell monolayer as described above.

### Quantitative analysis of cell migration and modeling

After initial cellular patterns were created, as shown in Fig. 1D, cell motility was measured by acquiring images at multiple locations every 20 minutes for 18 hours using an automated live cell microscope (Eclipse Ti, Nikon). When the cells entered into the micropatterns, cells initially at the moving front and following cells entering into the ECM micropatterns have unstable collective monolayer migration due to increased competition from diminishing area. However, once the cells fully entered the confined nanogrooved regions, the migration behavior of the cells became collectively stable. Therefore, for consistency we obtained measurements after cells had traveled 150 microns into the channels to capture stable monolayer migration behavior. The cell nucleus was tracked at each time point and custom computational routines were implemented in MATLAB 8.1 (The MathWorks, Natick, MA) to plot trajectory and calculate the migration speed and persistence time parameterized by a persistence random work model^54^,^55^,^56^. Migration speed was measured by calculating displacement per time. Individual cell speeds and persistence times from each experiment were averaged to obtain a single experiment’s parameter means and the associated standard errors. Each experiment was technically replicated at least three times with the number of 40–100 cells for each experiment. Associated error bars represent ±2 SE and were derived using standard propagation of error techniques. Finally, statistical analysis was carried out by student’s t-test for between two groups (MCF-10A vs. PIC3CA knockin cells, flat vs. nanogrooved), and two way ANOVA test followed by post-hoc (Duncan method) analysis for different pattern widths versus cell type or substrate type. P values less than 0.05 were regarded as statistically significant.

To implement the diffusion anisotropy model presented here, particles (representing cells) perform a random walk within geometrically constrained two-dimensional space (varying constraint in the y direction). Following random walk behavior a particle moves stepwise in a random direction. That is, at each time step (Δ*t*) the particle undergoes a displacement (*δ*) along a randomly chosen direction. Here the trajectory direction vector is limited so the x component is in the positive direction to account for constraints imposed by cells behind the leading edge in collective migration. Likewise, the particle displacement is constrained by restricting movement outside of the defined boundary. Similar, adjacent cells in a cell sheet can be viewed as a lateral constraint. At each time step, the displacement was evaluated to determine if it resulted in the particle crossing the boundary. If the particle crossed the boundary the displacement beyond the boundary was rejected. If the particle did not cross the boundary the next random walk displacement was implemented. Following 100,000 steps/particle averaged over 1,000 particles the mean displacement in the x direction was determined for varying widths of the migration pattern.

## Additional Information

**How to cite this article**: Nam, K.-H. *et al.* Multiscale Cues Drive Collective Cell Migration. *Sci. Rep.* **6**, 29749; doi: 10.1038/srep29749 (2016).
